# The Origin of Phenotypic Heterogeneity in a Clonal Cell Population In Vitro

**DOI:** 10.1371/journal.pone.0000394

**Published:** 2007-04-25

**Authors:** Daniel Stockholm, Rachid Benchaouir, Julien Picot, Philippe Rameau, Thi My Anh Neildez, Gabriel Landini, Corinne Laplace-Builhé, Andras Paldi

**Affiliations:** 1 GENETHON–Centre National de la Recherche Scientifique (CNRS), UMR 8115, Evry, France; 2 Ecole Pratique des Hautes Etudes, Paris, France; 3 Oral Pathology Unit, School of Dentistry, The University of Birmingham, Birmingham, England; Children's Hospital Boston, United States of America

## Abstract

**Background:**

The spontaneous emergence of phenotypic heterogeneity in clonal populations of mammalian cells *in vitro* is a rule rather than an exception. We consider two simple, mutually non-exclusive models that explain the generation of diverse cell types in a homogeneous population. In the first model, the phenotypic switch is the consequence of extrinsic factors. Initially identical cells may become different because they encounter different local environments that induce adaptive responses. According to the second model, the phenotypic switch is intrinsic to the cells that may occur even in homogeneous environments.

**Principal Findings:**

We have investigated the “extrinsic” and the “intrinsic” mechanisms using computer simulations and experimentation. First, we simulated *in silico* the emergence of two cell types in a clonal cell population using a multiagent model. Both mechanisms produced stable phenotypic heterogeneity, but the distribution of the cell types was different. The “intrinsic” model predicted an even distribution of the rare phenotype cells, while in the “extrinsic” model these cells formed small clusters. The key predictions of the two models were confronted with the results obtained experimentally using a myogenic cell line.

**Conclusions:**

The observations emphasize the importance of the “ecological” context and suggest that, consistently with the “extrinsic” model, local stochastic interactions between phenotypically identical cells play a key role in the initiation of phenotypic switch. Nevertheless, the “intrinsic” model also shows some other aspects of reality: The phenotypic switch is not triggered exclusively by the local environmental variations, but also depends to some extent on the phenotypic intrinsic robustness of the cells.

## Introduction

Phenotypic heterogeneity in genetically homogenous cell populations is frequently observed in *in vitro* cell cultures. Some cells within genetically uniform populations of various organisms such as bacteria or yeast exhibit striking phenotypic variability [1], [2]. In *in vitro* mammalian cellular systems the, spontaneous emergence of phenotypic heterogeneity is a rule rather than an exception. Phenotypic heterogeneity is systematically observed in cultures of various lines of genetically identical cells even in controlled environments. These different phenotypes can be strikingly different; for example, non malignant cells can spontaneously produce neoplastic subclones at a frequency dependent on the culture conditions. [3]–[6]. Conversely, cells in isogenic populations of malignant cell lines in culture can spontaneously revert to stable non-malignant phenotypes.[7], [8].

Since cell lines and primary cells are commonly used to investigate regulatory processes and gene expression during cell differentiation, understanding phenotypic differentiation in clonal populations is a fundamental problem in biology. There are two mutually non exclusive basic models for this phenomenon. In the first model the phenotypic switch is thought to occur as the consequence of extrinsic factors. Two initially identical cells may become different because they encounter different local environments that induce alternative adaptive responses. By changing its phenotype the cell itself contributes changes of the local microenvironment and thus elicits responses from the surrounding cells that are likely to lead to continuous dynamic changes in the population. According to the second model, the phenotype switch is intrinsic to the cells. The phenotypic changes therefore may occur even in an homogenous environment and may result from asymmetric segregation of intrinsic fate determinants during cell division that lead to the change in gene expression patterns.

These two hypotheses can be investigated in cell cultures. One example studied in our laboratory showed that two subpopulations appear spontaneously in proliferating C2C12 mouse myogenic cells [9]. The main population (MP) represents 95 to 99% of the cells and is characterized by a relatively low level of *Mdr1* activity. The remaining 5 to 1% of the cells have relatively high levels of *Mdr1* activity and constitute the so-called side population (SP). Transcriptome analyses showed distinct expression levels of several genes in the two cell subtypes [10] suggesting that the SP and the MP cells may represent different stages in the differentiation process. Phenotypically, the SP cells of the C2C12 cell line display a number of features reminiscent of stem cells (similarly to other SP cells which were identified by the same criteria of fluorescent dye exclusion in various cell lines or primary cell cultures). On the other hand, MP cells are more like the cells committed to differentiate into myotubes.

In the present study we investigated the “intrinsic” and “extrinsic” hypotheses of phenotypic differentiation using simple multi-agent computer modelling. In this approach each cell is considered as an autonomous “agent”. Only the rules determining the action of individual agents are defined, while the behaviour of the whole system emerges from the collective behaviour of all agents. The computer simulations showed that both the “intrinsic” and “extrinsic” models could in principle produce heterogeneous populations, but the spatial distribution of the different cell types was different across the two models. The predictions made by the models concerning the distribution of the different cell types were compared with the spatial distribution of the SP and MP cells in the C2C12 cell line. The results indicate that both models capture certain aspects of the *in vitro* situation, and at the same time underline the importance of the microenvironment and local intercellular interactions that may be related to phenotypic switching. .

## Results

We have designed a simple agent-based model to analyse the possible outcomes of the “extrinsic” and the “intrinsic” mechanisms of phenotypic differentiation. Agent-based models follow a bottom-up simulation strategy, defining simple rules that govern the behavior of individual agents (the cells, in our case) without a global view of the whole system. In the next sections we the describe the general model, followed by the intrinsic and extrinsic variants of the growth model.

### The basic parameters in the model

Our models are based on a limited number of simplified assumptions about how individual cells migrate, interact with each other, divide and die. These universal features were deduced from direct observations on real life cultures of myogenic C2C12 cells and their control values (i.e. model parameters) were chosen to represent observed realistic ranges.

**Figure 1:**
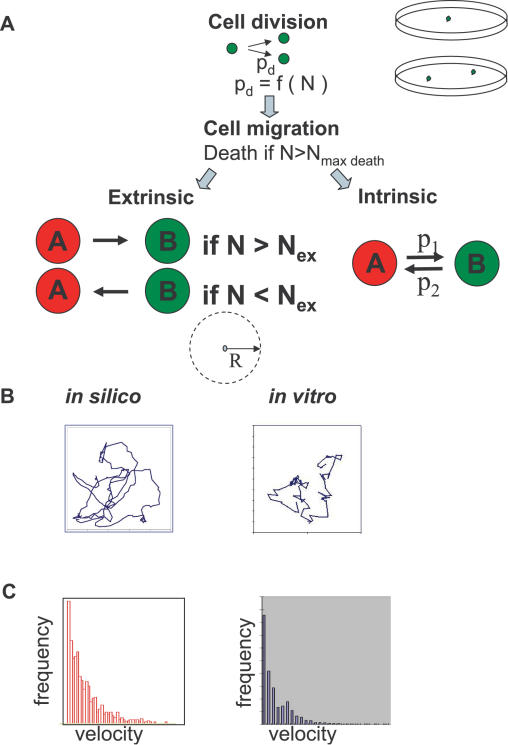
The basic parameters in the model. **A**: Multiagent computer simulation of the “extrinsic” and “intrinsic” mechanisms. Cells migrate and divide in the same way in the two models, and cell death is a function of the local cell density. The phenotypic switch of each cell is either dependent on the local cell density in the “extrinsic” (left) or a fixed probability in the “intrinsic” (right) model.” N” is the number of neighbours in the circle with a radius “R”; “p_d_” is the probability of division; p_1_ and p_2_ are the probabilities of the A and B type cells to change their phenotype in the “intrinsic” model; N_ex_ is the threshold, given by the number of neighbours. **B**: Characteristics of the cell migration in the computer model and *in vitro*, as observed in the cultures of the C2C12 cell line. The trajectories of a single cell simulated *in silico* (left) and its *in vitro* counterpart (right, determined by video microscopy), are shown. **C**: The exponential distribution of the cumulative velocity magnitudes in the simulation (left) and *in vitro*, as determined experimentally (right).

Since cell migration plays a crucial role in the model, the migration characteristics were defined on the basis of videomicroscopic observations of growing C2C12 cell cultures (more than 3000 cell velocity values over an 18h period). In accordance with earlier reports [11], [12] we found that cells migrate randomly (Fig. 1B) and that the cumulative velocity magnitudes (at a given time) as well as the velocities of the same cell over long periods, follow an exponential distribution (Fig. 1C). Therefore, the migration velocities of the simulated cells were generated using an exponential probability distribution function.

In the model, all cells divided at each iteration step, but the survival of the daughter cells depended on the local cell density. The highest local density, N_max death_, above which the daughter cell cannot survive (i.e. it dies) was defined as the number of neighbours within a circular region around the cell with radius R. The number of neighbours for each cell is therefore determined at each iteration step. In all simulations the arbitrary value of R = 1 was used-it is important to note that the actual value of R gains a meaning only in relation to the value of N_max death_ because the cells *in silico* have no size (i.e. they are represented by a point). As a result, the values of R = 1 and N_max death_ = D give similar results to R = 2 and N_max death_ = 4xD. In an early version of the model the cells always died when the value of N_max death_ was reached. This led to the formation of large empty patches (never observed in real cell cultures), so in order to make the model more realistic, we implemented cell death as a probabilistic event. The probability that a cell will die, p_death_ increases from 0 to 1 following a sigmoidal curve as a function of N_max death_. As a result, the excessive cell density variations in the virtual cell population disappeared. Although not confirmed by direct observation, the assumption that cell death will depend on the local cell density is intuitively easy to understand: cell density correlates with the gradient of nutrients, oxygen and toxic metabolites making less likely in dense regions than in sparse ones.

We explored the parameter space defined by the migration velocity and N_max death_. Values from 40 to100 cells/R for N_max death_ provided a cell density that approximated the observed maximal number of neighbours in a circle with a radius of 8 to 20 µm in real cultures of C2C12 cells (close to confluence). The average cell migration velocity emerged as a crucial parameter for the model. Low average velocity values (<0.2) led to the formation of small clusters of cells separated by empty strips. Because such patterns were never observed in real cultures and the real cell average velocity was higher than 0.2, we programmed velocity values between 0.3 and 1.0. The observed cell distribution pattern was random at all N_max death_ values, as estimated by the nearest neighbour distance method (see Materials and Methods).

The “extrinsic” and the “intrinsic” hypotheses for the generation of cellular heterogeneity were implemented varying the parameters defined above. We assumed the existence of two cell types: A and B. In both cases, the same rules for migration, division and death (as described above) were applied to type A (representing the SP cells) and B (representing the MP cells).Type A cells can differentiate into B cells and *vice versa* under conditions defined by the hypothesis under test (“intrinsic” *versus* “extrinsic”; Fig. 1A). In spite of the generality of the model, we focused our attention on a range of model parameters where the A cells are in minority, because in the experimental system used for the verification of the predictions in the second part of this work one cell type, the stem cell-like SP cells, represent only 1 to 2% of the whole population.

### The “intrinsic” model

**Figure 2:**
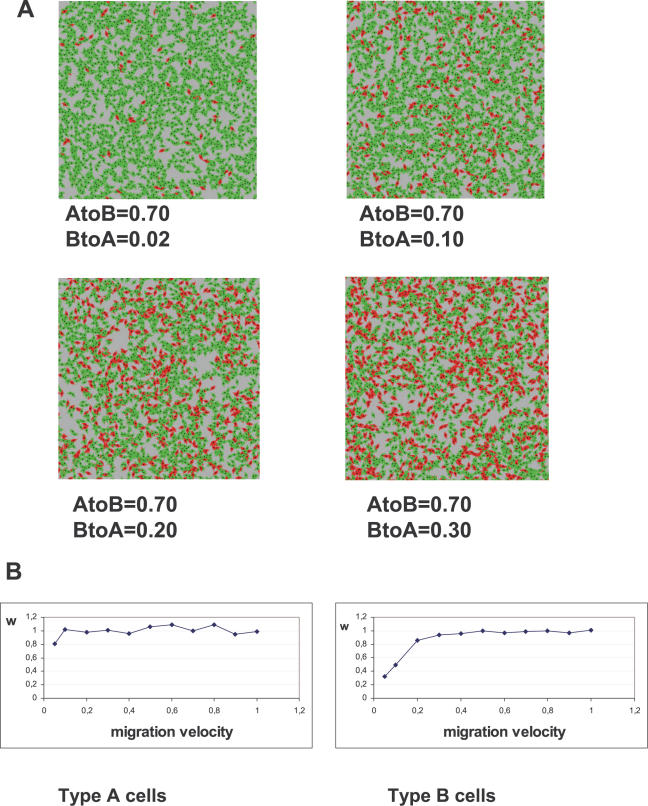
The “intrinsic” model. **A**: Results of the “intrinsic” model simulations for large values for p_BtoA_. Note corresponding increase in proportion of type A cells (in red) and their random distribution. **B**: Analysis of type A (left panel) and type B (right panel) cell distributions in the “intrinsic” model as a function of average migration velocity using the standardized nearest neighbour distance (*w*). If *w* = 1, the cells are randomly distributed. Small standardized nearest neighbour distances (*w*<1) indicate clustering; this is only observed for B cells with very low average migration velocities (<0.2). In these examples p_AtoB_ = 0.7 and p_BtoA_ = 0.02, but similar results were obtained for other values of p.

In the “intrinsic” model we addressed the question of cell autonomy of the phenotypic switch. At each simulation step, type A cells have a fixed probability p_AtoB_ to transform into B cells, and B cells have a probability p_BtoA_ to become A cells (the environment plays no role in the switching). The simulations start with a single A cell which replicates to fill the available space, reaching a maximum size at an equilibrium between growth and death. B cells appear with a frequency determined by p_AtoB_. We explored the effects of varying the values of p_AtoB_ and p_BtoA_ between 0 and 0.5. If p_BtoA_ = 0, B cells overgrow A cells and the whole population becomes B type. If B cells can switch to A (p_BtoA_≠0), the size of the two subpopulations, [A] and [B], reaches a steady state equilibrium with a ratio [A]/[B] that depends on p_BtoA_/p_AtoB_. (Fig. 2A). The spacial randomness of cell distribution was estimated by the calculation of the standardized nearest neighbour distance (*w*). The two cell types are distributed randomly in the population both during a) the growth phase and b) at the equilibrium when velocity values are higher than 0.2 (Fig. 2B). This suggests that the random element in cell type spatial distribution is a generic property of the “intrinsic” model.

**Figure 3:**
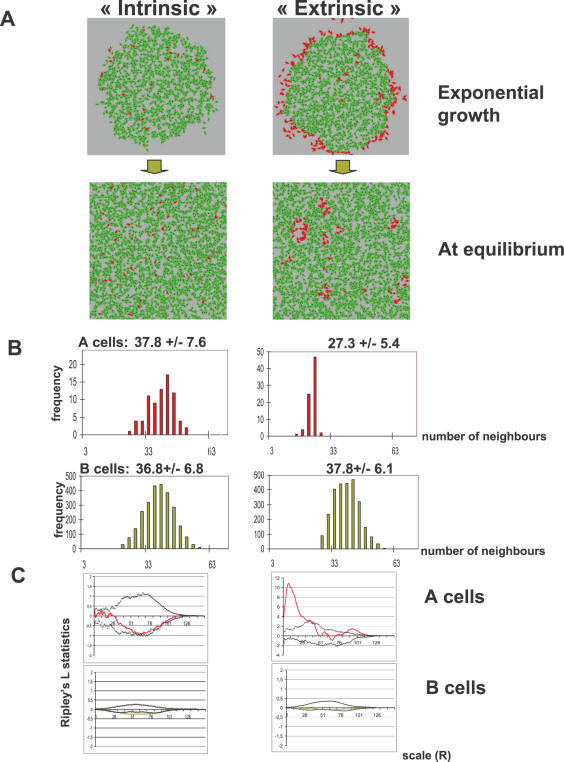
Results of a typical simulations. **A**: Results of a typical run at the exponential growth phase and at equilibrium (type A cells are red and type B cells are green). Left panels: “intrinsic” phenotypic switch; right panels: “extrinsic” phenotypic switch.. In all simulations N_max death_ = 40 was used. **B**: The distribution of the number of neighbours around the A and B cells (upper and lower panels, respectively) in the “extrinsic” (left) and “intrinsic” (right) models. The average number of neighbours and the standard deviation is indicated for each panel. **C**: Analysis of the spatial randomness of cell distribution at equilibrium using Ripley's L statistics. Ripley's L a point pattern with those generated by a homogeneous Poisson process. The plot shows the estimate of L(h) for various values of R ( = radius of a circle around the cell), and compares them to the line y = 0 (expected in a homogeneous Poisson process). An envelope defining the confidence interval is obtained from the maximum and minimum L(h) estimates of a large number of Monte Carlo simulations. The point set is significantly clustered in the range of scales where the estimated L(h) values are larger than 0 and lay outside the region defined by the envelope. This is the case for type A cells when considering small distances in the “extrinsic” model (red line). However, there is no significant clustering of type A cells (red line) in the “intrinsic” model and no clustering of type B cells (green line) in any of the two models, because all L(h) values are close to 0 and lay inside the region defined by the envelope (black lines).

We were most interested in the case where the ratio [A]/[B] is small, because it corresponds to the (real) cultured examined (see later). The Fig. 3A shows the results of a typical run, where we set p_AtoB_ = 0.70 and p_BtoA_ = 0.02. As a result of the high probability of A to B conversion and the relatively low probability of the B to A reversion, A cells become the rare phenotype, representing a small fraction of the population. Obviously, this is symmetrical to the case where p_AtoB_ = 0.02 and p_BtoA_ = 0.70. As indicated above, the A and B cells are distributed randomly at equilibrium (Fig. 3C). As expected, there was no significant difference in the number of neighbours between the A and B cells (Fig. 3B). The lack of significant clustering of the A and B cells was confirmed by the Ripley's L function analysis.

### The “extrinsic” model

In the “extrinsic” variant of the model, the phenotypic switch was programmed as a consequence of the change in the cellular microenvironment. Type A cells switch to type B if the number of neighbours in a circular region with a radius R around them is higher than a fixed number N_ex_. Type B cells switch to type A if the local density drops below the limit N_ex_. The local cell density, therefore, plays a role in the induction of the phenotypic switch correspond which is similar to cell survival: cell density here is also expected to correlate with the gradient of nutrients, secreted factors, oxygen, toxic metabolites, etc. Therefore, in our context, A and B cells represent two forms of phenotypic adaptation to high- and low density environments. However, we do not make explicit hypotheses concerning the precise mechanism underlying this phenotypic switch.

**Figure 4:**
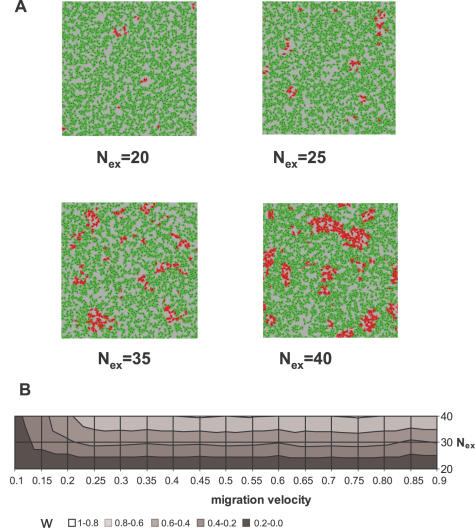
The “extrinsic” model. **A**: Simulations with the “extrinsic” model using increasing values of N_ex_. Note the increasing proportion and clustering of type A (red) cells with increasing N_ex_. In all simulations N_max death_ = 40 was used. **B**: Analysis of type A cell distribution as a function of average migration velocity and varying N_ex_ using the standardized nearest neighbour distance. Type A cells were clustered (*w*<1) at all but small average velocity values at all N_ex_ values analysed.

A typical simulation starts with a single A cell, with type B cells appearing first at the centre of the growing population (where the cell density is the highest). Local fluctuations in cell density due to random cell movement and cell death sometimes allow B cells to switch back to type A. During the growth phase, A cells are observed on the periphery of the population (Fig. 3A) but when the cells fill all the available space, the subpopulations reach a dynamic equilibrium with [A]/[B] determined by the N_ex_/R ratio (see Fig. 4 A). When A cells switch to B type at low density (during the equilibrium phase) the majority of the population is composed of B cells. However, if N_ex_ is close to N_max death_, the number of A and B cells is nearly equal. When compared to the “intrinsic” model, the spatial distribution of the cells in this “extrinsic” model is markedly different. The rare phenotype A cells typically form groups or clusters in areas with low local cell density for all tested values of N_ex_. The B cells are distributed randomly when N_ex_<N_max death_, but some clustering appears when N_ex_ approaches N_max death_ and the sizes of the two subpopulations become comparable (not shown). We also examined the effect of migration velocity on the spatial distribution of cells and found that clustering of A cells was not affected by this parameter (Fig. 4B). Therefore, cluster formation seems to be a generic feature of the “extrinsic” model and robust in the range of the parameters considered.

The results of a typical run are shown in Fig 2. The average number of neighbours around type A and type B cells (a measure of the local cell density) is significantly different (Fig. 3B). The distribution of neighbours around B cells is bell-shaped, while the distribution of neighbours around A cells is truncated above the value of N_ex_. While the non-random distribution of type A cells in the periphery of the exponential growth phase is evident, the clustering of A cells at the equilibrium phase was demonstrated by the standardized nearest neighbour distance method (*w*). If *w* = 1, the cells are randomly distributed, small standardized nearest neighbour distances (*w*<1) indicate clustering. The Ripley's test confirmed that A cells were significantly clustered, but the spatial distribution of the B cells was found random over all cell-cell distances (Fig. 3C).

### Experimental analysis

**Figure 5:**
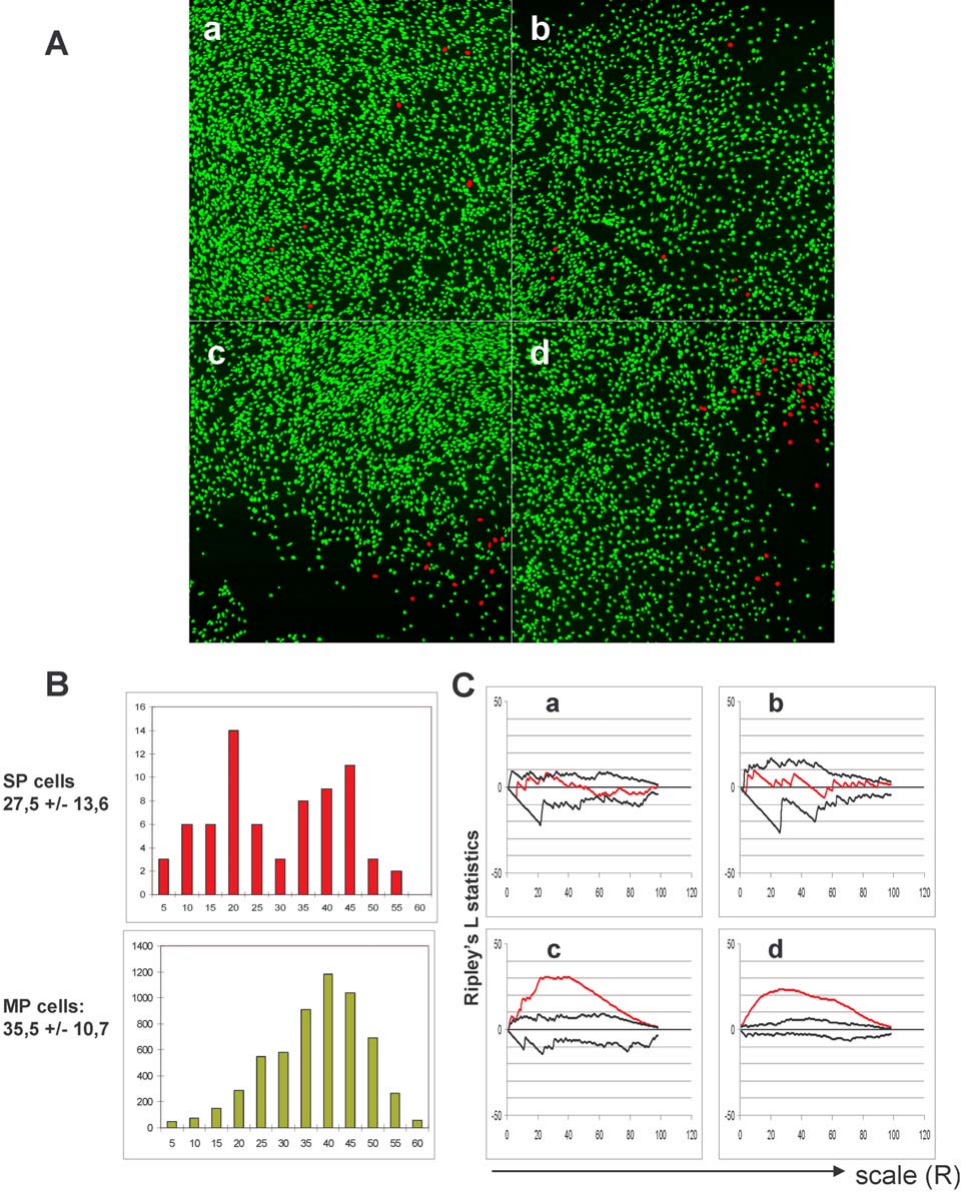
Localization of the SP cells in the growing population of C2C12 cells. **A**: The SP cells were identified on the basis of their capacity to exclude the fluorescent dye Hoechst 33342. Four representative images (*a, b, c* and *d*) are shown from regions with different cell densities. The nuclei are shown in false color (red for SP cells and green for MP cells). **B**: The frequency distribution of the number of neighbours is significantly different for SP and MP cells (upper and lower panel). The average number of neighbours is shown on the left side of each panel. Note the bimodal distribution for SP cells. The number of neighbours for each cell in a circle of R = 15 µm was calculated on the basis of the digitalized images. The two distributions were found to be significantly different (p<0.001) as analysed by the non-parametric Wilcoxon-Rank-Sum test. **C**: Analysis of the spatial randomness of SP cell distribution using Ripley's L statistics of the four images shown in Fig. 5A. The red lines indicate the L-functions for the SP cells over a range of *r = *100. The black lines show the upper and lower limits of the envelope functions for the images analysed. The *c* and *d* patterns are significantly different from a random pattern, because the values of the observed L-function are larger than the upper envelope function while the two other L-functions (panels a and b) indicate homogeneously distributed SP cells on the corresponding images.

The computer simulations show that both the “intrinsic” and “extrinsic” mechanisms are able to generate heterogeneous populations of cells with a stable proportion of the two cell types. The comparison of the “intrinsic” and “extrinsic” models provided testable predictions. Type A cells in the “extrinsic” model had on average fewer neighbours than B cells (Fig. 3B). Since the A to B switch was defined as a function of the local cell density, this was expected. An unforeseen consequence, however, was that the rare phenotype A tended to form clusters while the B cells are distributed randomly. The “intrinsic” model instead produced A cells distributed randomly and which had on average the same number of neighbours as B cells. These distinctive features in the two models was investigated experimentally using the C2C12 myogenic cell line. There are two subpopulations in the cultures of these cells: the rare stem-like SP (A cells in the model) and the myoblast-like MP cells (B cells in the model). The SP cells can differentiate into MP cells and *vice versa*. Analysis of the spatial distribution of the SP cells provides an experimental test for the model prediction, because clustering of the A cells in the “extrinsic” model was more apparent when the frequency of these cells was low. We then examined the distribution of the SP cells in cultures of C2C12 cells. To do this, cells were grown *in vitro* and stained using Hoechst 33342 dye in the culture dish. Instead of analysing the fluorescence intensity of the nuclei by cytometry, we acquired nuclear images by two photon microscopy. Since the size of the cell population was considerably large, images from separate fields were acquired and analysed as statistical samples of the whole population. The analysis was performed using image segmentation software (developed in our laboratory) which makes possible the automatic measurement of the fluorescence intensity of the nuclei while also recording the position within the culture. Since SP cells represent only a small fraction of the population, it is necessary to analyse large numbers of cells to find enough SP cells for statistical analysis. The blue and red Hoechst 33342 fluorescence intensity of 5900 cells was analysed. Only 71 (1.2%) were identified as SP cells within the limits defined by the usual criteria. The results are shown in Fig. 5A. Many SP cells formed small groups in the regions with relatively low cell density on the periphery of the growing cell population. However, numerous MP cells were also found in these low dense regions, indicating that low density *per se* may not be sufficient to generate the SP phenotype. On the other hand, many SP cells were found in the regions of high cell density. These cells did not form groups; they were dispersed in a high density MP environment (Fig. 3A). The number of neighbours around the SP cells had a bimodal frequency distribution (Fig. 5B), suggesting the existence of two distinct subpopulations. The frequency distribution of the neighbours (a measure of the local cell density) for SP and MP cells is significantly different (Wilcoxon-Rank-Sum test, p<0.001). In order to determine whether the spatial distribution of SP cells shows significant clustering, we used Ripley's L-statistics. Since the whole cell population in the culture-dish can not be analysed on a single image due to its large size, the analysis was done on separate sample images. Significant clustering was found only for SP cells in low density regions, while the distribution in the high density regions it did not significantly differ from the uniform pattern. On Fig. 5C we show two examples of statistics for clustering and two for homogeneous distributions of SP cells. Interestingly, the MP cells also show clustering over a wide scale of distances, suggesting that the randomization of the pattern by cell migration and death was incomplete in the analysed growing population.

### The “hybrid” model

**Figure 6:**
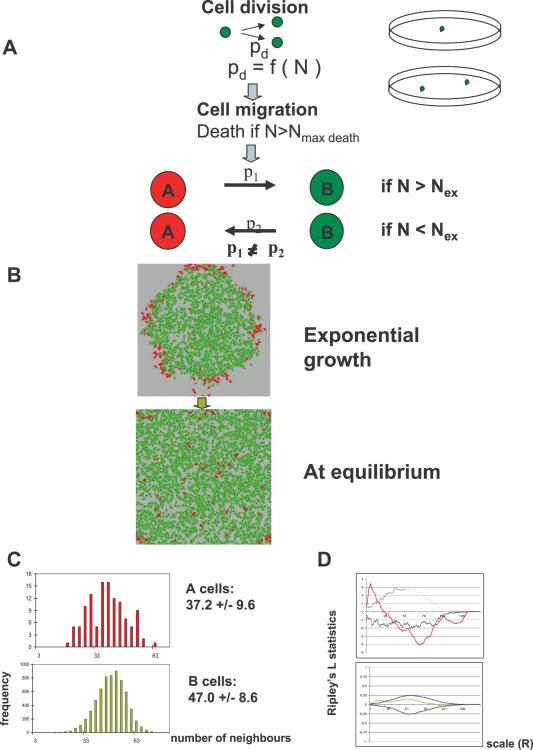
The “hybrid extrinsic-intrinsic” model. **A.** Cells migrate, divide and die under the same conditions as in the “extrinsic” and “intrinsic” models. The phenotypic switch of each cell is dependent on the local cell density as in the “extrinsic” model, but the cells encountering a favourable microenvironment undergo phenotypic change with probabilities p_AtoB_ and p_BtoA_. B: Results of a typical simulation of the “hybrid” model during the growth phase and at equilibrium. Note the simultaneous presence of small clusters and dispersed single type A cells. p_AtoB_ = 0.7 and p_BtoA_ = 0.4. C: The distribution of the number of neighbours around the A and B cells (left and right respectively) in the hybrid model. The average number of neighbours and the standard deviation are indicated for each panel. Note the more dispersed distribution of type A cell neighbours. D: Analysis of the spatial distribution randomness of SP and MP cells using Ripley's L statistics. The upper panel shows the type A cell L-function (red line) with values larger than 0 and outside the range defined by the upper-and lower-envelope functions (black line) (this indicates significant clustering of type A cells at small R distances). The type B cells (green line) are randomly distributed, because the L(h) values are close to 0 at all scales (R).

The dissimilarity in the distribution of the rare phenotype SP cells in culture and the type A cells in both the “extrinsic” and “intrinsic” models indicates that the phenotypic switch (at least in our system) may follow an intermediate scheme, where each model emulates reality only partially. One possibility is that low density *per se* is not sufficient to generate the SP phenotype in all cells because the MP and SP phenotypes are robust and are able to resist, to some extent, microenvironmental fluctuations. To test this assumption we designed a third “hybrid” model that combined the “extrinsic” and “intrinsic” assumptions. In the “hybrid” model the phenotypic switch is triggered by the microenvironment: type A cells can switch to the type B phenotype if they have more than N_ex_ neighbours (again, within a circular region of radius R). Type B cells can switch back only if the local cell density becomes lower than the limit N_ex_. However, the cells encountering a favourable microenvironment undergo phenotypic change with an intrinsic probability p_AtoB_ and p_BtoA_ , so a fraction of cells keep their original phenotype even in a permissive microenvironment (Fig. 6A). If p_AtoB_ and p_BtoA_ = 1, the model is equivalent to the “extrisic” version. The results shown in Fig. 6B were obtained by using N_ex_ = 30 values (the same as in Fig. 3A) and the p values were fixed at p_AtoB_ = 0.7 and p_BtoA_ = 0.4. Although the distribution of the number of neighbours is no longer bimodal, the simulations show a type A cell neighbour distribution (Fig. 6C) reminiscent of that observed for the SP cells. This could be interpreted as an indication that the “hybrid” model matches reality better.

### Primary myoblast culture

**Figure 7:**
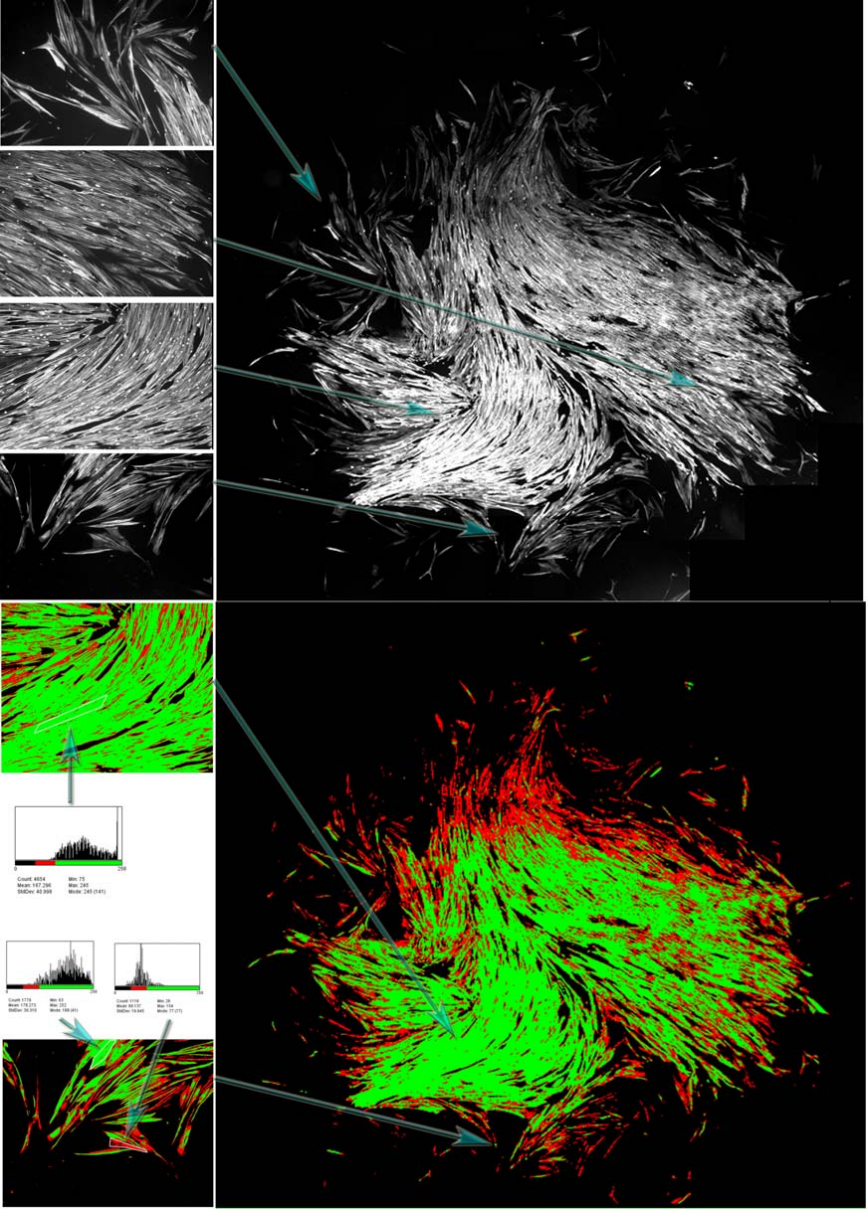
Clonal population derived from a human primary myoblast. **A**: The cells were fixed and immunostained with an anti-desmin antibody. A series of 55 pictures were obtained and compiled into a single picture showing the whole population. The variation in the intensity of desmin immunostain is higher at the periphery of the growing population. The left panel shows 4 high resolution images of different parts of the population pointed by the blue arrows. **B**: Colour coded image of the same population as on the A panel. The colour code is based on the intensity of the pixels (red: low, green: high intensity). On the left are shown 2 high resolution images with the same color code. In the upper left, a region of interest is indicated in white with the corresponding pixel histogram shown beneath. Two additional histograms show the pixel intensities of two regions of interest: high-(green) and a low-desmin expressing cells (red). Note that the low desmin expressing cells are more frequent at the periphery of the culture.

In order to clarify whether the non-random distribution of the rare phenotype cells is observed only in the C2C12 line or whether it is a general feature, we analysed clonal populations of primary human myoblasts. These were obtained by cloning of individual cells from a primary myoblast (muscle biopsy) culture. The cultures were allowed to grow to several hundred cells, fixed and immunostained with an anti-desmin antibody (a muscle-specific intermediate filament protein and one of the earliest markers of activated muscle precursor cells). Desmin is present in all myoblasts, but its expression level depends on the degree of commitment of the cell to the myogenic differentiation path [13]. This marker is frequently used to isolate pure myoblast populations [14]. A typical isogenic population is shown on Fig 7. Although no accurate quantitative measures were performed, it is obvious that the level of the desmin protein was highly variable within the cluture. Cells with the lowest expression levels (presumed to be less engaged in differentiation) were observed preferentially on the periphery. These observations show that phenotypic heterogeneity can also emerge spontaneously in a clonal population of primary cells and the uneven distribution of the different cells is reminiscent of the results obtained using the C2C12 cell line.

## Discussion

The basic model presented in this paper simulates the growth of a clonal cell population and provides a simple framework for testing the “intrinsic” and “extrinsic” hypotheses on the origin of phenotypic heterogeneity. The values of the parameters controlling cell motility, cell cycle length and cell death were deduced from observations on living cell cultures and were the same in both the “intrinsic” and the “extrinsic” versions. One could call into question the assumption that both A and B cell types follow the same rules of proliferation and migration. However, the opposite case, i.e. different proliferation and/or migration behaviour, would be more restrictive in the context of our model because it would be based on the assumption that proliferation and migration differences are part of the phenotypic differences between the two cell types. The results presented here show that even in the more general and parsimonious case of identity of proliferation and migration, phenotypic heterogeneity in a clonal cell population is possible. Differences in these characteristics between the two cell types would act to reinforce the heterogeneity.

We have examined two different hypotheses for the origin of phenotypic heterogeneity by computer simulation and experimental analysis. Computer simulations show that in the “extrinsic” model, the rare A phenotype cells were clustered in regions of low local cell densities, whereas the “intrinsic” model predicted random distribution of all cells in the cell culture.

According to the “extrinsic” model, cell differentiation depends on external cues. In the “intrinsic” model the spontaneous phenotypic switch occurs in a cell autonomous way with a given probability where intracellular interactions do not play a role. In the “intrinsic” model, since the phenotype switch is a relatively rare event, each cell type produces two daughter cells of the same type. Whether these cells remain close to each other depends on cell migratation. Random cell migration acts as a homogenizing factor that can disrupt clusters formed by cells of the same lineage. However, it also acts as the source of local heterogeneity of cell density. This constitutes a source of variability in the immediate environment of each cell such as the number of cell to cell contacts, the establishment of local concentration gradients of nutrients, oxygen or molecules secreted by the cells. Hence, in the “extrinsic” model random cell migration locally promotes the phenotypic switch.

The higher incidence of the A type cells in the low density regions in the “extrinsic” model was expected, because the phenotypic switch was conditioned by the low number of neighbours. However, clustering of the A cells in the “extrinsic” model and even distributions of these cells in the “intrinsic” model could not be predicted, based on the model's initial conditions, because the key parameter, cell migration, is stochastic. Clustering of the A cells is, therefore, a distinctive key prediction that could be analysed experimentally. It is worth noting, that in our models stable subpopulations of A and B type cells are maintained only if the phenotypic switch is reversible.

A computer simulation model of a complex phenomenon such as cell differentiation is inevitably general and based on a number of simplifications. On the other hand, an experimental system is always unique, so the experimental testing of the predictions of an abstract model must reconcile these two facets. The C2C12 cell line appeared as a good compromise for the experimental analysis of the clustering prediction. Reversible differentiation of the rare stem-like SP cells into myoblast-like MP cells and *vice versa* has been described [9]. The SP–MP transformation is a true differentiation step, because both cell types differ in their morphology, gene expression pattern and biological characteristics [9], [10]. Therefore, the A cells in the simulation share basic characteristics with the SP cells and the B cells are similar to the MP cells.

The observation that the SP cells had the tendency to be grouped in regions of low local cell density is consistent with the “extrinsic” model and stresses the importance of the local microenvironment in the initiation of the phenotypic switch. This conclusion was corroborated by the observation of position-dependent emergence of phenotypic heterogeneity in a population of primary myoblasts. Nevertheless, the “intrinsic” model also captures a part of reality, because rare phenotype cells were also found dispersed in regions of high cell density. Therefore, the “intrinsic” and “extrinsic” mechanisms are likely to correspond to two idealized solutions that act together. This is confirmed by the “hybrid” model, which provides a phenotype distribution in the population similar to that observed in the real cell culture.

What is the biological meaning underlying the “intrinsic” and “extrinsic” characteristics? We think that the “extrinsic” model refers to the cell's capacity to react to fluctuations in the environment by changing its gene expression pattern conditioned by the local concentration gradients of factors and metabolites secreted by the cells, nutrients, oxygen etc. However, the phenotypic change is not a continous transition initiated automatically by the variation of the local environment, rather it is like switch of a multistable system from one stable state to another. The cellular phenotype is robust and can resist small stochastic variation of the environment which can only induce fluctuations around the stable phenotypic state. Large fluctuations, however, destabilize the cell and generate changes. In this sense, the “intrinsic” probability in the hybrid model refers to the phenotypic robustness based on epigenetic mechanisms and transcription regulation networks rather than to a spontaneous propensity for differentiation. As a consequence, adaptation of a cell to the local microenvironment may constitute the first step in the emergence of a new cellular phenotype. Cell-to-cell interactions could then act to preferentially stabilize the new state. As a result, the cell type composition of the originally homogenous population becomes heterogeneous and tends to a steady-state equilibrium, in spite of the fact that the phenotype of each individual cell may vary. Stochastic processes are usually considered as a deleterious noise, but as it was suggested earlier [15], [16], they can play a positive role in the process of differentiation. Our observations also provide support for this view: the population level stability is based, on one hand, on the stochastic fluctuation of the cells induced by local microenvironment and, on the other hand, on the stabilizing forces of cell interactions.

Current conceptual models tend to abandon the classical assumption of a strict hierarchy during differentiation and understand cell differentiation as a dynamic process [17]. For example, Kaneko and co-workers proposed on the basis of modelling studies that the differentiation of the cells is realized through an “isologous diversification” process, a mechanism by which identical state cells can diversify through the interplay between internal dynamics and external interactions [18]. Another modelling study has also demonstrated that auto-stabilization of stochastic processes by interdependence of cells for proliferation is essential for differentiation and pattern formation [19].

Although our experiments were carried out on *in vitro* cell cultures, our results may contribute to the better understanding of *in vivo* processes also. Differentiation of various cell types from a stem cell pool is a typical example of the process of phenotypic diversification [20]. The importance of the local cellular environment and cell-to-cell communication is increasingly recognized in normal differentiation and in neoplastic transformation [21]. Terms like “niche” borrowed from ecology are more and more used to describe the impact of tissue context and microenvironment on cell differentiation *in vivo* [22]. Our results suggest that phenotypic differentiation and niche formation are tightly linked and interdependent processes; both emerge from the multitude of local interactions between the participating cells. A practical consequence of this interdependence is the inherent difficulty in establishing cultures of cells with homogenous phenotype.

## Materials and Methods

### Analysis of cell migration velocity

Records of cell migration were done using a Zeiss Axiovert 100M confocal microscope. C2C12 cells were maintained under controlled CO_2_ atmosphere and temperature. Bright field images were recorded every 10 minutes for 18 hours. Image acquisition was done at high resolution (1024×1024 pixels) with aid of Zeiss LSM 510 software for PC. Migration velocities were calculated with a “Manual Tracking” plug-in (Fabrice Cordelières, Institut Curie, Orsay, France) of ImageJ software (http://rsb.info.nih.gov/ij/).

### Computer simulations

The computer simulation was performed using the Netlogo language, specifically designed to make simple agent-based models (Wilensky, U. 1999. NetLogo. http://ccl.northwestern.edu/netlogo/. Center for Connected Learning and Computer-Based Modeling, Northwestern University. Evanston, IL). The migration, division and death parameters were implemented identically in the “extrinsic” and “intrinsic” models. To implement cell migration, we defined the migration velocity as the linear distance the cell moves from its position in each step of the simulation and calculated this distance for each cell on the basis of an exponential probability distribution function determined from experimental observations. The direction of migration was considered to be random. The cells are allowed to divide at each iteration step. The probability of cell death, p_death_ is calculated for each cell after the division. A value N_max death _and a standard deviation, SD of N_max death_ is defined before each run for the maximal number of neighbours in a circle with a radius R. The value of p_death_ increases from 0 to 1 as a function of this parameter following a sigmoidal curve. As a result, the distribution of the life lengths of the cells follows a Gaussian distribution.

The total size of the population was determined by the sum of the divisions and deaths. Although no rule was defined for growth of the whole population, it followed a typical logistic kinetics and the maximal density of the cell population in any circular area with a radius R of the virtual culture dish oscillates around the value of N_max death_.

### Cell cultures

C2C12 cells were obtained from the American Type Culture Collection (CRL 1772) and routinely propagated in proliferation Dulbecco's Modified Eagle's Medium (DMEM, Gibco BRL) with 4,5 g/ml of glucose, supplemented with 20% (v/v) foetal calf serum (FCS, Hyclone), 100 U/ml penicillin and 100 µg/ml streptomycin. The cultures were grown at 37°C under a humidified atmosphere of air with 7% CO2. Initial plating density was between 2 .10^3^ and 3.10^3^ cell/cm^2^ and the cells were cultured for 6 days. 35 mm glass bottom culture dishes (Mat Tek Corporation) were used for cell culture and two photon analyses.

The primary human mononuclear cells from muscle biopsies were obtained from the Genethon's cell bank and cultured in Skeletal muscle cell growth medium (Promocell–C-23060) supplemented with 10% calf serum (PAA–A15-043), glutamax (Gibco–35050.038) and 50 µg/ml gentamicine (Gibco–15751.037). Individual cells were cloned by cell sorter (MoFlo–Dako) and allow to expand in a colony of several hundred cells.

### Phenotype detection

The C2C12 cells were stained by the DNA dye Hoechst 33342 (Sigma-Aldrich B-2261) at a final concentration of 11 µg/ml for 90 min at 37°C under mild shaking. The controls were incubated in the presence of 100 µM of verapamil (Sigma V-106 batch 28 H4699). The images were collected by a two-photon microscope under conditions of Hoechst excitation and processed by a software [23] that allows the automated segmentation of the nuclei, the measurement of the fluorescence intensity of each nucleus and the recording of their position in the plate. The cells with a given fluorescence intensity can then be located easily on the recorded image of the culture plate for further analysis.

The colonies of primary cells were fixed with pure methanol (for 60 min at−20°C) and immunostained by an anti-desmin monoclonal antibody (1/40-Sigma–clone DE-U-10 Product n°D1033).

### Statistical analysis

The number of neighbours around each cell in the simulations and the cell cultures was calculated automatically on the basis of the digitalized images and the distributions of the frequencies were compared by two sided t-test. The results were confirmed by the non-parametric Wilcoxon-Rank-Sum test.

The standardized nearest neighbour distance method was used to describe the degree of spatial clustering of the A and B cell distributions obtained in the “extrinsic” and “intrinsic” models. This method uses the average distance from every cell to its nearest neighbour to determine if the cells are clustered, distributed homogenously or dispersed. The standardized nearest neighbour distance, *w*, is calculated as the ratio of the average nearest neigbour distance (W) and the expected nearest neighbour distance if the cell distribution were homogenous (E[W]): *w* = W/E[W]. (W is calculated as the average of all distances to the nearest point from any point *i*. The expectation of the nearest neighbour distance of point s under the hypothesis of complete spatial randomness is a function of point density: E[W] = 1/√λ , where λ is the cell density.) If *w* = 1, the cells are randomly distributed. Small standardized nearest neighbour distances (w<1) indicate clustering and *w*>1 indicates dispersion.

In order to test the significance spatial clustering of the cells in the simulations and in the cell cultures the Ripley's L-function was used [24]. It was computed using the software program Programita kindly provided by Thorsten Wiegand. Ripley's L function provides a summary of spatial dependence over a wide range of scales of pattern, including all event-event distances, not just the nearest neighbor distances. It is used to compare a point pattern with point patterns generated by known processes, e.g. a homogenous Poisson process. The test gives a plot of the estimate of L(h) at different values of R ( = radius of a circle around the cell), and compares this to the line y = 1 expected under a homogenous Poisson model. An envelope defining the confidence interval and obtained from the maximum and minimum L(h) estimates of a large number of Monte Carlo simulations allows a simple visualisation of the range of scales where clusters are observed.

## References

[1] Avery S (2005). Cell individuality: the bistability of competence development.. Trends in Microbiology.

[2] Ko E, Yomo T, Urabe I (1994). Dynamic clustering of bacterial population.. Physica D.

[3] Rubin H (1993). 'Spontaneous' transformation as aberrant epigenesis.. Differentiation.

[4] Rubin H (1993). Cellular epigenetics: effects of passage history on competence of cells for "spontaneous" transformation.. Proc Natl Acad Sci U S A.

[5] Rubin H (2001). Selected cell and selective microenvironment in neoplastic development.. Cancer Res.

[6] Rubin H (2005). Degrees and kinds of selection in spontaneous neoplastic transformation: an operational analysis.. Proc Natl Acad Sci U S A.

[7] Lavrovsky VA, Guvakova MA, Lavrovsky YV (1992). High frequency of tumour cell reversion to non-tumorigenic phenotype.. Eur J Cancer.

[8] Sun C, Antonionio RJ, Redpath JL (1996). Reversion of UVC-induced tumorigenic human hybrid cells to the non-tumorigenic phenotype.. Eur J Cancer.

[9] Benchaouir R, Rameau P, Decraene C, Dreyfus P, Israeli D (2004). Evidence for a resident subset of cells with SP phenotype in the C2C12 myogenic line: a tool to explore muscle stem cell biology.. Exp Cell Res.

[10] Decraene C, Benchaouir R, Dillies MA, Israeli D, Bortoli S (2005). Global transcriptional characterization of SP and MP cells from the myogenic C2C12 cell line: effect of FGF6.. Physiol Genomics.

[11] Czirok A, Schlett K, Madarasz E, Vicsek T (1998). Exponential distribution of locomotion activity in cell cultures.. Physical Review Letters.

[12] Mombach J, Glazier J (1996). Single cell motion in aggregates of embryonic cells.. Physical Review Letters.

[13] Li H, Choudhary SK, Milner DJ, Munir MI, Kuisk IR (1994). Inhibition of desmin expression blocks myoblast fusion and interferes with the myogenic regulators MyoD and myogenin.. J Cell Biol.

[14] Webster C, Pavlath GK, Parks DR, Walsh FS, Blau HM (1988). Isolation of human myoblasts with the fluorescence-activated cell sorter.. Exp Cell Res.

[15] Kupiec J (1983). A probabilist theory for cell differentiation, embryonic mortality and DNA C-value paradox.. Specul Sci Technol.

[16] Paldi A (2003). Stochastic gene expression during cell differentiation: order from disorder?. Cellular and Molecular Life Sciences.

[17] Loeffler M, Roeder I (2004). Conceptual models to understand tissue stem cell organization.. Current Opinion in Hematology.

[18] Furusawa C, Kaneko K (2006). Morphogenesis, plasticity and irreversibility.. Int J Dev Biol.

[19] Laforge B, Guez D, Martinez M, Kupiec JJ (2005). Modeling embryogenesis and cancer: an approach based on an equilibrium between the autostabilization of stochastic gene expression and the interdependence of cells for proliferation.. Prog Biophys Mol Biol.

[20] Acklin SE, van der Kooy D (1993). Clonal heterogeneity in the germinal zone of the developing rat telencephalon.. Development.

[21] Soto AM, Sonnenschein C (2004). The somatic mutation theory of cancer: growing problems with the paradigm?. Bioessays.

[22] Moore K, Lemischka I (2006). Stem cells and their niches.. Science.

[23] Benchaouir RP, Greppo J, Rameau N, Stockholm PH, Garcia1 D (2006). Combination of quantification and observation methods for study of ”side population”cells in their “in vitro” microenvironment.. Cytometry A;.

[24] Ripley B (1977). Modelling spatial patterns (with discussion).. J. Roy. Stat. Soc. B..

